# Molecular Phylogenetic Analysis of the *Stegana ornatipes* Species Group (Diptera: Drosophilidae) in China, with Description of a New Species

**DOI:** 10.1673/031.011.0120

**Published:** 2011-02-23

**Authors:** Jin-Ming Lu, Tong Li, Hong-Wei Chen

**Affiliations:** ^1^ Department of Entomology, South China Agricultural University, Tianhe, Guangzhou, 510642 China; ^2^ Key Laboratory of Zoological Systematics and Evolution, Institute of Zoology, Chinese Academy of Sciences, Datunlu, Chaoyang, Beijing, 100101 China

**Keywords:** Drosophilidae, molecular phylogeny, mitochondrial DNA, *Stegana ornatipes* group, Oriental region

## Abstract

A new species of the *Stegana* (*Steganina*) *ornatipes* species group (Diptera: Drosophilidae) is described from Hainan, China, *S*. (*S*.) *xipengi* sp. nov. Based on the mitochondrial *ND2* and *COI* gene sequences, the relationships among eight species from mainland China of the *ornatipes* group, and their relationships to the *undulata, nigrolimbata* and *shirozui* species groups of the same subgenus, are investigated, using two species of the subgenus *Stegana, S. emeiensis* and *S. quadrata*, as outgroups. The result shows that *S*. (*S*.) *mengla* is debarred from the *ornatipes* group.

## Introduction

So far five species groups have been identified in the subgenus *Steganina* Wheeler (Diptera: Drosophilidae) of the genus *Stegana* Meigen: *coleoptrata* group (Laštovka and Máca 1982; Chen and Chen 2008), *nigrolimbata* group (Sidorenko 2002; Cao and Chen 2008), *shirozui* group (Chen et al. 2009), *undulata* group (Sidorenko 2002) and *ornatipes* group (Cheng et al. 2009), and they included 51 species; most of them were from the Oriental region except for some species of the *coleoptrata* group from the Palearctic region. The *ornatipes* group includes ten species from the Oriental region: *S*. (*S*.) *vietnamensis* Sidorenko, 1997 from Virtnam; *S*. (*S*.) *albiventralis* Cheng, Gao et Chen, 2009; *S*. (*S*.) *angusigena* Cheng, Gao et Chen, 2009; *S*. (*S*.) *chitouensis* Sidorenko, 1998; *S*. (*S*.) *lingnanensis* Cheng, Gao et Chen, 2009; *S*. (*S*.) *mengla* Cheng, Gao et Chen, 2009; *S*. (*S*.) *nulliseta* Cheng, Gao et Chen, 2009; *S*. (*S*.) *ornatipes* Wheeler et Takada, 1964; *S*. (*S*.) *pilosella* Cheng, Gao et Chen, 2009 and *S*. (*S*.) *zhaofengi* Cheng, Gao et Chen, 2009 from China. This group is supported by the following morphological characters as the diagnosis: surstylus large, with a strong prensiseta apically and several thin, long setae; 10th sternite mostly narrowed, nearly arcuate, with a pair of projections posterolaterally; gonopods with a pair of projections sublaterally. On the other hand, this group is similar to the *nigrolimbata* group in sharing the following morphological characters: palpus mostly black, sometimes yellow basally; gena yellow to brown, narrow (ch/o ≤ 0.10); aedeagus basally contiguous to aedeagal apodeme; which shows the both are more closely related to each other than other members of the subgenus *Steganina*.

Recently, some studies of molecular phylogeny were appeared to the subfamily Steganinae (Otranto et al. 2008; He et al. 2009a, b; Zhao et al. 2009; Li et al. 2010). Otranto et al. (2008) reconstructed the phylogenetic relationships among 13 species of 8 genera of Steganinae based on the DNA sequences of the cytochrome oxidase subunit I (*COI*) gene, however, in their phylogenetic analysis, only two *Stegana* species were sampled as the representative. Li et al. (2010) investigated the phylogenetic relationships among seven of the Chinese species of the subgenus *Stegana* (s.s.) based on the DNA sequences of the NADH dehydrogenase subunit 2 (*ND2*) gene, using two species of the subgenus *Steganina* (*S. nigrilimbata* Duda, 1924 and *S. ctenaria* Nishiharu, 1979) as outgroup taxa.

In the present study, we described a new species of the *ornatipes* group from Hainan, China. We also constructed the molecular phylogeny based on the mtDNA sequences of *ND2* and *COI* genes. To investigate the relationships in *ornatipes* group and with the other species groups of subgenus *Steganina*, we employed the additional seven species from mainland China of this group and *S. nigrolimbata* Duda, *S. xiaoleiae* Cao and Chen, *S. ctenaria* Nishiharu, and *S. undulata* de Meijere which belong to *nigrolimbata, shirozui* and *undulata* species groups of the subgenus *Steganina* as ingroup taxa Two species from subgenus *Stegana, S. emeiensis* Sidorenko and *S. quadrata* Cao and Chen were chosen as outgroup taxa.

## Materials and Methods

All materials were collected on tussock and tree trunks along streams in forest, preserved in 75% ethanol immediately and identified (Table 1). A small piece of tissue was removed from the fly abdomen and used for the DNA extraction; then, the body and terminalia parts were dried and deposited in the Department of Entomology, South China Agricultural University, Guangzhou, China (SCAU). McAlpine (1981) was followed for morphological terminology and Zhang and Toda (1992), and Chen and Toda (2001) for the definitions of measurements, indices and abbreviations.

### DNA extraction and sequencing

The total DNA was extracted using the DNA extraction Kit (TIANGEN®) according to the manufacture's protocol. The *ND2* gene and the 5' end of *COI* gene were amplified. Primers used were given in table 2. The PCR cycle program comprised an initial 3 min of predenaturation at 94 °C, 35 cycles of amplification ( 50 s of denaturation at 94 °C; 1 min of annealing at 53 °C for *ND2*, 49 °C for *COI*; 1 min of extension at 72 °C), and a final elongation for 5 min at 72 °C. When possible, purified amplified products were directly run on an ABI 3730 sequencer for sequenceing, otherwise they were cloned into the pMD18-T plasimid vector (TAKARA®), and then sequenced. The related *ND2* sequences of *S. emeiensis, S. quadrata, S. ctenaria* and *S. nigrolimabata* were retrieved from the National Center for Biotechnology Information (NCBI); the related *COI* sequences of *emeiensis, S. nigrolimbata* and *S. undulata* were also retrieved from the NCBI.

**Table 1.**
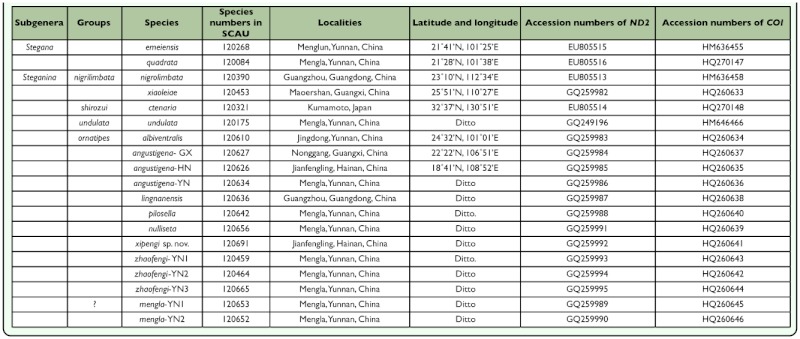
Collecttion data of samples for DNA sequencing, and accession numbers of the *ND2* and *CO1* sequences.

### Phylogenetic analyses

The sequences were aligned by the Clustal W (Thompson et al. 1994) method implemented in program MEGA 4.0 (Tamura et al. 2007) with default options. A partition homogeneity test (PHT) between the *ND2* and *COI* sequences was performed with PAUP 4.0b10* (Swofford 2002). The program DAMBE 5.0.80 (Xia and Xie 2001) was used to measure the nucleotide substitution saturation using the method of Xia et al. (2003) as the substitution saturation masked the phylogenetic signal (Lopez et al. 1999; Philippe and Froterre 1999). Base compositions were investigated by means of the software PAUP 4.0b10* (Swofford 2002), and a χ^2^ test was also used to test the nucleotide composition homogeneity. Uncorrected pairwise divergence was estimated by program MEGA 4.0 (Tamura et al. 2007).

Phylogenetic trees were constructed by using the maximum parsimony (MP) and maximum likelihood (ML) in PAUP 4.0b10* (Swofford 2002), the Bayesian inferring (BI) method performed in MrBayes 3.2.1 (Huelsenbeck and Ronquist 2001; Ronquist and Huelsenbeck 2003). The MP and ML trees were searched by the heuristic method, with initial trees obtained by randomly adding taxa, and the TBR algorithm was used in branching swapping. Branch support for each node in the MP and ML trees was assessed by 1000 bootstrap replicates. The nucleotide substitution models of ML and BI analyses were selected by MrModeltest 2.3 (Nylander 2004) using the hierarchical likelihood ratio test (hLRT) criterion (Posada and Crandall 1998). In the BI analyses, the site-specific models were assigned to dataset partitioned by locus (2 data partitions) and by codon positions (6 data partitions). Two independent runs with 2,000,000 generations were implemented in parallel, sampling frequency of every 100 generations was employed. When the average deviation of split frequencies fell well below 0.01, the two runs were stopped. For each running, the 5,000 early-phase samples were burn-in, the rest samples were used in summarizing and a majority rule tree showing all the compatible partitions was obtained.

**Table 2.**
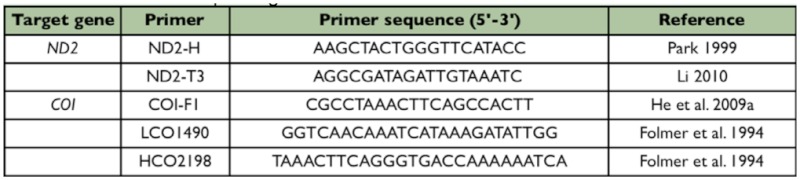
Primers used for PCR and sequencing.

Partition Bremer support (PBS) was used to show the contribution of each gene partition to the Bremer support of the simultaneous analysis (Baker and DeSalle 1997). Values can be positive, negative or zero and sum of all the partitioned Bremer support values at a node will equal the Bremer support value for that node. A positive PBS value suggests support for the node by that gene, whereas a negative PBS value indicates that the partition lends conflict to a given node, and zero indicate that the partition lends neither support nor conflict to a given node. The partitioned Bremer support values were calculated using the partitioned constraint file in TreeRot v3 (Sorenson 1999).

## Results

**Figures 1–2.**
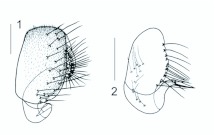
*Stegana* (*Steganina*) *xipengi* sp. nov., ♂: 1. Epandrium, cercus and surstylus (lateral view); 2. surstylus (frontal view). Scale bars = 0.1 mm. High quality figures are available online.

***Stegana* (*Steganina*) *xipengi* sp. nov. (Figures 1,2)**
**Diagnosis**
  This species is related to *S*. (*S*.) *albiventralis* from Yunnan in having the entirety white katepisternum, but clearly distinguishable from it by the palpus yellow basally, black distally, the mesonotum brown, without stripe (in *albiventralis*: palpus entirely yellow; mesonotum brown, with yellow stripe medially).
**Description**
  Male: Frons and face not rectangular in profile. Eyes red. Ocellar triangle black, with 1 pair of small setae above ocellar setae.
  Postvertical setae slightly behind vertex ridge. Frons shiny, brown, with sporadic, minute setulae submedially, and a black, transverse band above ptilinal fissure. Proclinate orbital setae slightly nearer to ptilinal fissure than to inner vertical setae. Pedicel brown; first flagellomere yellow only basally, mostly black. Face black with yellow, transverse band medially, broadened ventrally; facial carina absent. Clypeus black medially, yellow laterally. Palpus yellow basally, black distally, with 1–2 longer setae distally and several shorter setae basally. Gena yellow, narrow. Vibrissa prominent; other orals small. Occiput glossy, yellow, but black around occipital foramen. Mesonotum brown. Mesopleuron with a black longitudinal stripe above (running from propleuron to base of halter). Postpronotal lobe brown on upper part, white on lower part, with 1 long and a few short setae. Acrostichal setulae approximately in 10 irregular rows. Prescutellar setae 1 pair. Katepisternum entirely white. Scutellum brown; basal setae divergent; apical setae crossing with each other. Wing dark brown anteriorly, pale posteriorly, curved downward on distal part. Basal medial-cubital crossvein present. C_1_ with 2 isometric setae. Costal vein with 9 minute spinules on ventral surface between veins R_2+3_ and R_4+5_. Vein R_2+3_ obviously curved to costa at tip; Veins R_4+5_ and M_1_ convergent distally. Halters white basally, greyish brown distally. Legs whitish yellow, brown on apical part of fore femur, and fore and hind tarsomeres, dark brown to black on medially on mid and hind femora, with 2 dark brown rings on fore and mid tibiae. Fore femur with 3–4 setae on distal part of ventral surface. Apical seta present on mid tibia. Preapical dorsal setae present on all tibiae. Mid tibia (misused to mid tarsus in Cao and Chen 2008; Cheng et al. 2009) with 5 strong setae on basal part of dorsal surface. Mid and hind tarsomeres with 2 and 1 row(s) of minute cuneiform setulae on ventral surface, respectively; fore and hind 1st tarsomeres slightly shorter than the rest combined; mid 1 st tarsomere longer than the rest combined. Abdominal all tergites dark brown. Sternites brown; 3rd to 5th broadened; 6th covered with 5th. Epandrium pubescent except for anteroventral margins, with approximately 21 setae near posterior margin on each side (Figure 1). Cercus separated from epandrium, setigerous, lacking pubescence (Figure 1). Surstylus separated from epandrium, with several thin, long setae on inner margin and surface (Figure 2), apically strongly curved and with 1 strong prensiseta (Figure 2). The hypandrium, gonopods, aedeagus and aedeagal apodeme were lost when clearing them in KOH solution.
**Measurements**
  BL = 2.76 mm in holotype; ThL = 1.32 mm; WL = 2.58 mm; WW =1.12 mm. Indices: arb = 8/7, avd = 0.83, adf = 1.20, flw = 1.80, FW/HW = 0.36, ch/o = 0.08, prorb = 1.16, rcorb = 0.82, vb = 0.30, dcl = 0.40, presctl = 0.60, sctl = 1.80, sterno = 0.90, orbito = 2.20, dcp = 0.20, sctlp = 1.00, C = 1.86, 4c = 1.22, 4v = 1.74, 5x = 1.40, ac = 9.33, M = 0.61, C3F = 0.66.
**Type**
  Holotype: ♂ (SCAU, No. 120589), CHINA: Jianfeng, Ledong, Hainan, 18°41′N, 108°52′E, alt. 750 m, 14.iv.2008, *ex* tussock, X.P. Chen.
**Etymology**
  Patronym of the collector Xipeng Chen (SCAU).
**Distribution**
  China (Hainan).

**Table 3.**
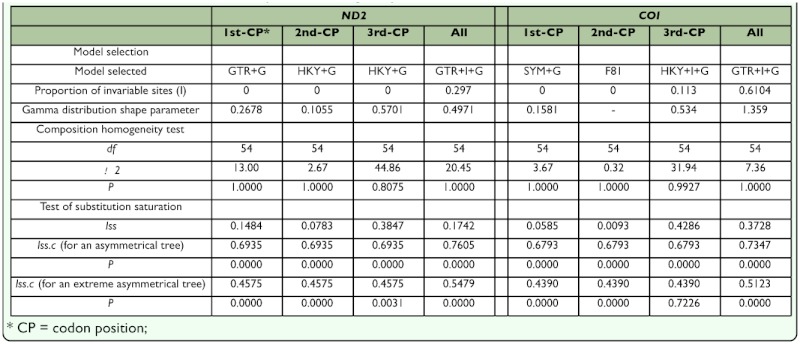
Results of model selection, composition homogeneity test and test of substitution saturation.

### Molecular analysis Data set analysis

The alignment was 1739 nucleotide positions (1029 for *ND2* and 710 for *COI*, respectively) in length. There were end gaps in the *ND2* sequence of *S. undulata* (sites 1–22) and in the *COI* sequence of *S. xiaoleiae* (sites 1–33). The base composition of *ND2* and *COI* were generally AT rich with a mean of 83% and 69%, respectively. It contained high AT contents in the 3rd (94.2% and 94.2%, respectively) codon positions. Performance of the Chi-square test was showed in table 3. It yielded a homogeneous base composition in the *ND2*-alignments and *COI*-alignments or in the separate condon positions of the two mitochondrial genes.

The test of substitution saturation showed that the observed index of substitution saturation (*Iss*) for *ND2*-alignments or for *COI*alignments was significantly lower than the corresponding critical index substitution saturation (*Iss.c*), indicating that there was little saturation in our sequences. However, when considering partitions separated by codon, we identified substitution saturation in the third codon position of the *COI*- alignments [*Iss* = 0.4286 < *Iss.c* = 0.4390 (for an extreme asymmetrical tree, p = 0.72)] (Table 3). Since none of the resulted trees of the present study are extremely asymmetric, there should be little substitution saturation in our sequence.

Table 4 shows the uncorrected pairwise p-distances for the *ND2* and *COI* sequences. The genetic divergence of *ND2* sequences of species within the *ornatipes* group ranged from 5.28% to 14.24%, and genetic divergence of *COI* sequences ranged from 4.28% to 10.49%, however, when we took no account of the *S. mengla*, the upper limits would declined to 8.37% and 8.42% for *ND2* and *COI*, respectively. Within the *ornatipes* group, divergences between *S. mengla* and other species ranged from 9.36% to 15.64% for *ND2* and from 8.57% to 10.93% for *COI*, whereas the genetic variance among groups ranged from 11.65% to 14.34% for *ND2* and from 8.57% to 11.23% for *COI*.

### Phylogenetic analysis

The PHT resulted in a p value of 0.062, indicating that no significant incongruence was found between the *ND2* and *COI* data sets. The best-fit models selected for the ML reconstruction and Bayesian inference were listed in table 3.

**Table 4.**
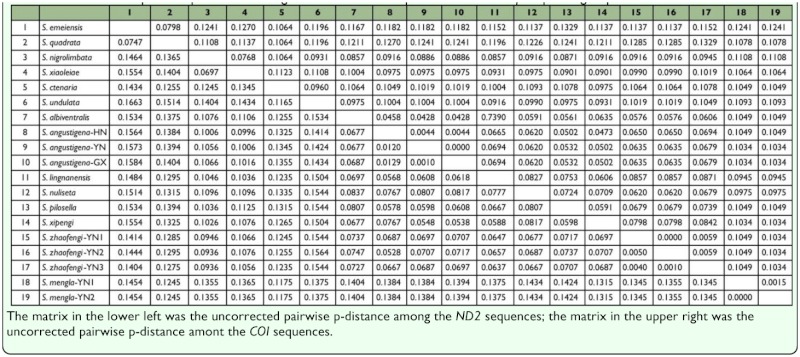
Uncorrected pairwise p-distance among the *ND2* and *COI* sequences of the *ornatipes* species group.

**Figure 3.**
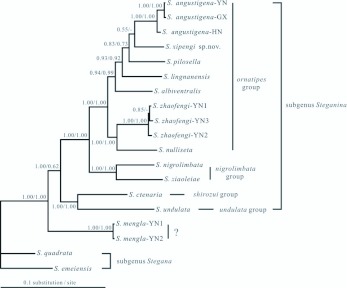
Bayesian tree of the *ornatipes* group deduced from the *ND2* and *COI* sequences. The numbers above the branches show the posterior probabilities of the corresponding node in the Bayesian inference. High quality figures are available online.

**Figure 4.**
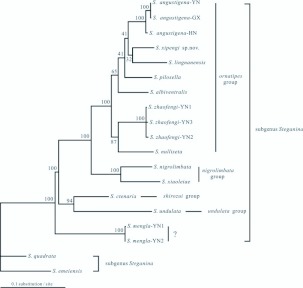
ML tree of the *ornatipes* group deduced from the *ND2* and *COI* sequences. The numbers above the branches show the bootstrap percentages (BPs) of the corresponding node in the ML analysis (-lnL = 7925.69531). High quality figures are available online.

**Figure 5.**
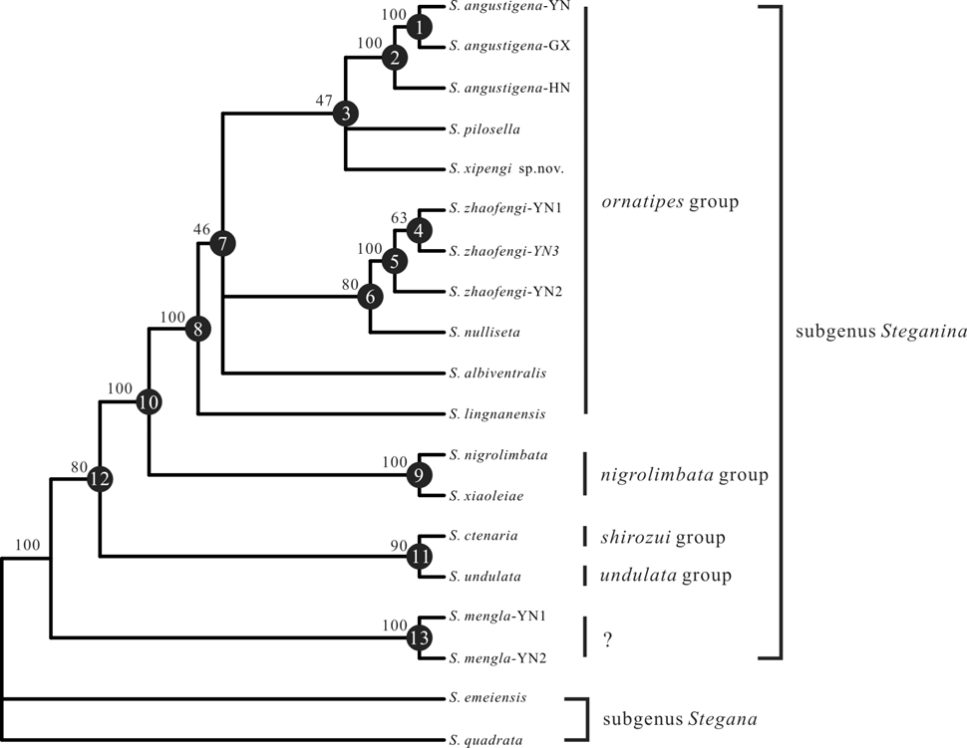
Strict consensus tree of two most parsimonious trees of the *ornatipes* group deduced form the ND2 and *COI* combined sequences. The numbers above the branches show the bootstrap percentages (BPs) of the corresponding node in the MP analysis [tree length = 1225, consistency index (Cl) = 0.6008, retention index (Rl) = 0.6362]. High quality figures are available online.

The relationships within the *ornatipes* group were not stable revealed by different treebuilding methods as the low supports for the basal nodes (Figures 3, 4 and 5), but it was surprising that *S. mengla* was debarred from the *ornatipes* group in all trees, and it was placed at the most basal clade of subgenus *Steganina* receiving great supports (MP BP, or bootstrap percentages of the MP analysis = 100; ML BP = 100; PP or posterior probability of the 2-/6-partition Bayesian inferring = 1.00/1.00). The remaining species of the *ornatipes* group were recovered as a monophyletic group with robust supports in all trees (MP BP = 100; ML BP = 100; PP = 1.00/1.00 in the 2-/6-partition Bayesian analyses, respectively). The *nigrolimbata* group appeared to be the closest relative to this monophyletic group with well supports (MP BP = 100; MLBP = 100; PP = 1.00/1.00) (Figures 3, 4 and 5). The Bayesian analysis yielded a general topology (Figure 3), which was mostly congruent with the result of the ML analysis (Figure 4). The monophyletic group diverged into two branches. One consist of *S. zhaofengi* triple and *S. nulliseta*, and the other further diverged into *S. albiventralls, S. lingnanensis, S. pilosella, S. xipengi* and *S. angustigenai* triple orderly in the Bayesian tree, whereas *S. pilosella* diverged prior to *S. lingnanensis* (ML BP = 41), leaving *S. lingnanensis* and *S. xipngi* as sister group in the ML reconstruction, but with a low support (ML BP = 32). The MP tree (Figure 5) differed from the ML and Bayesian tree at several points. It suggested a very basal position for *S. lingnanensis* in the *ornatipes* group and *S. xipengi* clustered with *S. pilosella* which was consistent with the Bayesian analysis. The Yunnan (-YN), Guangxi (-GX) and Hainan (-HN) samples of *S. angustigena* clustered together with well support (MP BP = 100; ML BP = 100; PP = 1.00/1.00), and so did in the YN1, YN2 and YN3 samples of *S. zhaofengi* (Figures 3, 4 and 5).

To determine the relative contributions of the two data partition to the combined analysis tree, partition Bremer supports were calculated and given in table 5. Support for combined analysis phylogeny came from *ND2* was a little bit more than that from *COI*. Nodes 1, 3, 6, 7 and 12 showed a mixture of positive and negative PBS scores.

## Discussion

The phylogenetic trees showed that the *ornatipes* group clearly appeared to be paraphyletic. To eliminate the effect of individual difference, another sample of *S. mengla* was included in the analysis, but the situation did not change. In morphological viewpoints, the *S. mengla* holds the same diagnostic characters of the *onartipes* group, which contradict with our molecular phylogeny as it formed a separate branch in the phylogenetic tree. The amount of genetic divergences between *S. mengla* and other species within the *ornatipes* group were high and overlapped to some extent with the divergence between species groups. Although speculative, the morphological convergence should be the reason for this situation. The convergent morphological evolution seems to be common in the subfamily *Steganinae* (Otranto et al. 2008), which is similar to the suggestion made in this research concerning convergent morphological evolution in *S. mengla*. Considering the closer relationship of *S. mengla* with the outgroup *S. emiensis* respect to the other species showed in the phylogenetic tree, it is possible that *S. mengla* is the interim species of the divergent between subgenus *Steganina* and subgenus *Stegana*. Of course, this hypothesis should be proved with analysis of suitable species of both the subgenus *Steganina* and subgenus *Stegana*. Except the *S. mengla*, the branch consist of the rest species of the *ornatipes* group showed the closer relationship with the *nigrolimbata* group than other species groups of subgenus *Steganina* was consistent with the morphological affinity in the two groups (Cao and Chen 2008).

**Table 5.**
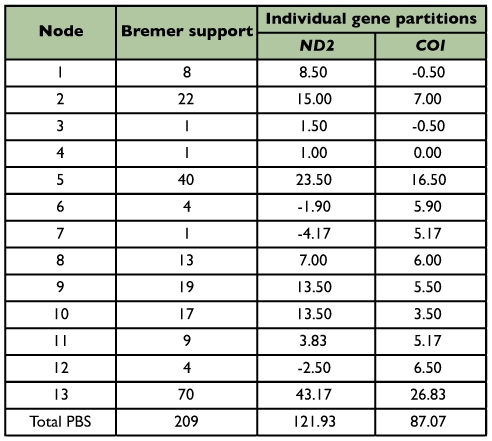
Partition Bremer support for all nodes in the MP tree (Fig. 5).

In general, the NADH dehydrogenase subunit genes are rapidly evolving, but the cytochrome oxidase subunit is more slowly evolving (Simon 1994). It was supposed that the *ND2* gene was better than the *COI* gene suited for species-level analysis, but the PBS analysis indicating that the contribution to the MP reconstruction in this research of the *ND2* gene was nearly the same as the *COI* gene. Our PBS analysis had implications for the conflicts of the two genes at some nodes (e.g., nodes 1, 3, 6, 7 and 12), suggesting that the two partitions data (*ND2* and *COI*) may be favoring an alternative tree topology. The relationships of these nodes should be viewed cautiously.

The genetic distances between Yunnan, Guangxi and Hainan samples of *S. angustigena* [p-distance of *ND2* = 0.0129 (GX vs. -HN), 0.0120 (-HN vs. -YN), 0.001 (GX vs. -YN); p-distance of *COI* = 0.0044 (GX vs. -HN), 0.0044 (-HN vs. -YN), 0.0000 (-GX vs. -YN)] were among the mean intraspecific variability of Meier et al. 2008 (1.3 ± 1.6%) for Diptera. In addition, no essential morphological character was found to distinguish the specimens of these three samples, indicating that they should be taken as conspecific ones. It was the same as the case of the YN1, YN2 and YN3 samples of *S. zhaofengi.* The genetic data [p-distance of *ND2* = 0.0050 (-YN1 vs. -YN2), 0.0040 (YN2 vs. -YN3), 0.0010 (-YN1 vs. -YN3); p-distance of *COI* = 0.0000 (-YNl vs. -YN2), 0.0059 (-YN2 vs. -YN3), 0.0059 (-YN1 vs. YN3)] also indicated the conspecific status of the three samples of *S. zhaofengi*.

Some relationships within the *ornatipes* group were not well resolved, especially the alternative placement of *S. lingnanensis, S. xipengi* and *S. pilosella*. Therefore, it may be worthy to increase either the genetic markers (such as nuclear markers) or the number of samples in the future phylogenetic analysis of the *ornatipes* group.
